# Native Kidney Renal Cell Carcinoma in Renal Allograft Transplant Patients – Our Experience

**DOI:** 10.15586/jkcvhl.v11i2.283

**Published:** 2024-05-14

**Authors:** Pavan Survase, Shashank Agrawal, Abhishek Singh, Ravindra Sabnes, Mahesh Desai

**Affiliations:** Department of Urology, Muljibhai Patel Urological Hospital, Nadiad, Gujarat, India

**Keywords:** immunosuppression, renal cell carcinoma, transplant

## Abstract

The immunosuppression administered to renal transplant recipients to safeguard renal function elevates their susceptibility to renal cancer, which is estimated to be 15 times higher than in the general population. The current study aimed to analyze various aspects of native kidney renal cell carcinoma (RCC) in renal transplant recipients. This study involved a retrospective analysis of 11 patients who underwent nephrectomy for RCC in native kidneys among renal transplant recipients at our institution since 1992. Our institutional incidence was 0.4%. Median age at presentation was 57 (49–60) years. The ratio of male: female was 10:1. Most patients were asymptomatic at presentation and native kidney disease before transplantation was undetermined. In our study, the median time interval between diagnosis of RCC and transplant was 9.1 (8.4–11.2) years. All patients underwent native kidney nephrectomy. Clear cell type was more common than papillary type, 3.5 (2.5–4.2). Ten patients were diagnosed with stage I disease and one patient had stage IV disease. Fuhrman nuclear grading revealed low grades in nine patients and three patients had Grade 3. Immunosuppressive therapy modification was done in nine patients. Meticulous follow-up of renal transplant patients is essential for earlier diagnosis and appropriate treatment of native kidney RCC in transplant recipients. Authors recommend every year follow-up in transplant recipients with special emphasis on ultrasound of native kidney.

## Introduction

The immunosuppressive therapy administered to renal transplant recipients to maintain renal function elevates their susceptibility to renal cancer, which is estimated to be 15 times higher than that of the general population (1). Studies have shown that the occurrence of renal cell carcinoma (RCC) among renal transplant recipients is 4.6%, contrasting with the 2% rate observed in the general population (2). Few studies demonstrated the incidence to be even higher. Hence, a thorough knowledge and consensus about the clinicopathologic characteristics of this well-known entity is essential for a transplant surgeon to offer comprehensive care to transplant recipients. The aim of this study is to assess the prevalence, management, and prognosis of native kidney RCC among renal transplant recipients.

## Methodology

This observational study involved a retrospective analysis of data collected from our institution’s database starting from 1992. Institutional Review Board approval was obtained for data collection and analysis. Patients diagnosed with RCC in the native kidney post-transplantation were included. Among the 2839 transplants performed at our institution until January 2020, 11 patients subsequently developed RCC in the native kidney.

Given the observed increase in the incidence of post-transplant RCC, our institutional protocol mandates annual screening ultrasonography of native kidneys for renal transplant recipients. Suspicious lesions detected during screening were further evaluated using contrast-enhanced computed tomography (CECT) of the abdomen and pelvis, with additional blood investigations and chest X-rays.

All patients suspected of having RCC in the native kidney underwent laparoscopic radical nephrectomy. Tumor staging was determined based on the 8th edition of the UICC TNM staging system, and tumor grading was assessed using the Fuhrman nuclear grading system established in 1982. Statistical analyses were performed using univariate analysis and SPSS software.

## Results

In the present study among the 11 patients who developed post-transplant native kidney RCC, 90.9% (10 patients) were males and 9.1% (1 patient) were females. Median age at presentation was 57 (49–60) years. Clinical and demographic details are mentioned in Table 1. Among the 2839 transplants conducted at our institution, 11 patients developed RCC, resulting in an incidence rate of 0.4%. Of these cases, 54.5% presented with right-sided tumors, while 45.5% had left-sided tumors, with no instances of bilateral disease observed.

**Table 1:** Clinical and demographic data.

Data		Number (percentage)
**Age (years) (Median, IQR)**		57 (49–60%)
Gender	Male	10 (90.9%)
	Female	01 (09.1%)
Laterality	Right	06 (54.5%)
	Left	05 (45.5%)
	Bilateral	0
Comorbidity	HTN	08 (72.7%)
	DM	02 (18.2%)
Presenting complaint	Asymptomatic	09 (81.8%)
	Painless gross hematuria	01 (09.1%)
	Weakness	01 (09.1%)
Native kidney disease before transplantation	Undetermined	06 (54.5%)
	Chronic sclerosing glomerulonephritis	03 (27.3%)
	Chronic tubulo-interstitial disease	01 (09.1%)
	Diabetic nephropathy	01 (09.1%)
Median time interval between initiation of dialysis and renal transplant (years) (Median, IQR)		5 (3–7)
The median time interval between transplant to the diagnosis of tumor (years) (Median, IQR)		9.1 (8.4–11.2)

A total of 72.7% of patients who were diagnosed with post-transplant native kidney RCC were hypertensive, and 18.2% of them had associated diabetes mellitus. 81.8% were asymptomatic at presentation and the tumor was picked up during the regular follow-up visits, one patient presented with weakness and one patient presented with painless gross hematuria. In 54.5% of patients, the native kidney disease was undetermined before transplantation, 27.3% of patients had chronic sclerosing glomerulonephritis, one patient had chronic tubule-interstitial disease and one patient had diabetic nephropathy. The median time interval between diagnosis of RCC in the native kidney post-transplant was 9.1 years.

All patients underwent laparoscopic radical native kidney nephrectomy; one patient underwent bilateral native kidney nephrectomy as he had a cortical cyst on the other kidney which was benign on HPE. Adrenal adenoma was noted in another patient hence adrenalectomy was performed along with tumorous native kidney nephrectomy.

On histopathological examination, 54.5% of patients had clear cell RCC and 45.5% had papillary type of RCC. 54.5% of patients had lesions in the upper pole of the kidney. The lesion in the middle and the lower pole was noted in 27.3% and 18.2% of patients, respectively. Pathological descriptions of the renal masses are depicted in Table 2.

**Table 2:** Pathological characteristics of renal mass.

Data		Number (percentage)
Location of RCC in the kidney	Upper pole	06 (54.5%)
	Middle pole	03 (27.3%)
	Lower pole	02 (18.2%)
Median tumor size (cm) (Median, IQR)		3.5 (2.5–4.2%)
HPE-Cell type	Clear cell RCC	06 (54.5%)
	Papillary RCC	05 (45.5%)
Tumor staging	pT1aN0M0 (Stage I)	06 (63.7%)
	pT1bN0M0 (Stage I)	02 (18.2%)
	pT4N0M0 (Stage IV)	01 (09.1%)
Fuhrman nuclear grading	I	04 (36.4%)
	II	05 (45.4%)
	III	02 (18.2%)

All patients received immunosuppressive therapy with either mycophenolate mofetil, cyclosporine A, tacrolimus, Azathioprine, prednisolone, or a combination of these. The induction agents used were daptomycin and antithymocyte globulin. Table 3 presents comprehensive information regarding the immunosuppressive agents administered, along with any modifications made following the nephrectomy of the tumorous native kidney.

**Table 3:** Immunosuppressive agents offered in patients post-transplant and modification done after tumorous native kidney nephrectomy.

Patient number	Post-transplant immunosuppression	Duration (years)	HPE	Size (cm)	Modification after nephrectomy
1	Tac, MMF, P	3	CRCC	1.8	E, Tac, MMF, P
2	Cy A, Aza, P	14	CRCC	3.2	Cy A, P
3	Tac, MMF, P	4	PRCC	2.5	Tac, MMF, P
4	Induction: Daptomycin Aza, MMF, P	3	CRCC	1.8	Aza, P
5	Cy A, Aza, P	7	PRCC	3	Aza, Tac, P
6	Azathioprine, MMF, Prednisolone	13	CRCC	4.2	Aza, MMF, P, E
7	Tac, MMF, P	4	CRCC	3.5	S, Aza, P
8	Tac, MMF, P	14	PRCC	4	MMF, P
9	Cy A, Aza, P	17	CRCC	5.2	Aza, P
10	Tac, MMF, P	19	PRCC	3.5	Aza, P
11	Induction: ATG Tac, MMF, P	4	PRCC	6	Tac, MMF, P

## Discussion

Carcinomas of the skin and lips, RCC, hepatocellular carcinomas, and lymphoproliferative disorders are the common forms of neoplasms post-transplantation. Urinary system malignancy precisely RCC is the second most commonly encountered after those of the skin (3). Excess risk is correlated to the obligation of offering immunosuppressive therapy to prevent rejection of the transplanted kidney. As previously stated, the incidence of RCC in renal transplant recipients is 4.6% (2). Multiple studies concluded a higher incidence. In our study, the incidence of RCC in renal transplant recipients was approximately 0.4% (taking into account the patients lost in follow-up). A comparison of our demographic data with other studies revealed similar results as given in Table 4.

**Table 4:** Comparison of demographic data with other similar studies.

Parameter	Present study	Alvarez et al.(6)	Filocamo et al.(7)	Gigante et al.(8)
Mean age	54.9 ± 6.4 years	48.5±11.1 years	48.1 years	53 years
Male:Female ratio	10:1	0.8:1	2.3:1	3.2:1

The majority of patients enrolled in our study presented asymptomatically, aligning with findings observed in studies conducted by Filocamo et al. (4) and Ianhez et al. (5). A study by Doublet et al. (6) reported a 3.9% incidence of RCC among 129 kidney transplant recipients screened with routine ultrasound of native kidneys. Filocamo et al. (4) suggested routine screening ultrasonography every 3 years, whereas, in our institution, renal transplant recipients undergo yearly reviews.

In our study, three patients presented with chronic sclerosing glomerulonephritis, one with chronic tubule interstitial disease, and another with diabetic nephropathy before transplantation, while the specific underlying condition of the remaining patients’ native kidneys was undetermined. On an outlook, patients with chronic kidney disease face an increased risk of developing neoplasms. Proposed risk factors include age, prior exposure to substances like tobacco and alcohol, sun exposure, and hereditary factors, as well as the etiology of kidney disease, duration of dialysis, and uremia-related immune dysfunction (6). Furthermore, the likelihood of RCC is heightened after renal transplantation, primarily due to immunosuppression, in addition to other contributing factors such as the reactivation of viruses with oncogenic potential, age at transplantation, and the duration since transplantation (7). Filocamo et al. (4) conducted a study involving 10 patients who developed RCC post-transplantation. Their findings revealed a spectrum of native kidney diseases among these individuals, encompassing hepatitis C virus-related cryoglobulinemic glomerulonephritis, glomerulonephritis, membranous proliferative glomerulonephritis, diabetic nephropathy, nephroangiosclerosis, and IgA nephropathy, each identified in one patient, while four patients had unspecified underlying diseases (9). In our study, the median time interval between diagnosis of RCC and transplant was 9.1 (8.4–11.2) years. Likewise, Echer et al. (8) observed a mean time interval of 10 years. Substantial evidence from various studies consistently indicates a considerable duration between the diagnosis of RCC and transplantation. This underscores the critical necessity for vigilant and timely follow-up care for all transplant recipients.

According to Filocamo et al. (4), RCC in renal transplant recipients tends to exhibit a more aggressive course compared to the general population, often leading to a discouraging prognosis and resistance to treatment. Numerous studies affirm that tumor size upon diagnosis significantly influences disease progression, with smaller tumors associated with a more favorable prognosis. Recognizing the favorable outcomes linked to early intervention, timely diagnosis of RCC is imperative. At our institute, the median tumor size upon diagnosis was 3.5 cm.

The distribution of cellular subtypes of RCC in our study cases mirrored that reported by Ianhez et al. (5), with clear cell carcinoma being the most prevalent followed by papillary carcinoma. Additionally, tumors located in the upper pole were more prevalent than those in the lower and mid poles. Most valid prognostic factors for RCC, include tumor size, TNM classification, and Fuhrman nuclear grading (8).

The five-year survival rate varies significantly depending on the TNM stage, ranging from 90 to 100% in stage I (T1N0M0), to less than 10% in stage IV. All of our patients were diagnosed with stage I disease, except for one who presented with stage IV disease. Renal cell carcinomas (RCC) are further categorized based on the Fuhrman nuclear grade into low (grades I and II) and high (grades III and IV) grades. In our study, the tumors were predominantly small and confined to stage I, with the exception of one patient in stage IV. Regarding Fuhrman nuclear grading, the majority of our patients exhibited low grades (I or II), with only two patients classified as grade II.

In our study cohort, oncological surveillance comprised biannual contrast-enhanced computed tomography (CECT) of the abdomen and pelvis, along with chest X-rays during the initial 2 years, followed by annual assessments for the subsequent 3 years. Among the six patients who completed the five-year follow-up, the cancer-free survival rate was 83.3%, with one patient succumbing to the disease. The remaining five patients have not yet completed the full five-year follow-up period. Notably, among these, two patients have achieved a 100% cancer-free survival rate at the three-year mark.

Despite the increasing occurrence of cancer among kidney transplant recipients, there is a paucity of data regarding the optimal management of immunosuppression following a cancer diagnosis. A comparative randomized trial assessing low-dose cyclosporine against regular dose revealed no disparity in graft survival or function. However, the low-dose regimen exhibited a lower incidence of malignant disorders and a higher frequency of rejection episodes (10). An additional randomized controlled trial involving 489 kidney transplant recipients, with a follow-up period of 20 years, demonstrated that both azathioprine and cyclosporine-based treatment regimens carried comparable long-term risks of cancer (11).

Yang et al. (12), in their subgroup analysis, found that the reduction of both mycophenolate and calcineurin inhibitors was associated with worsening graft function and lower graft survival. Recently, a consensus panel stated that the decision to withdraw calcineurin inhibitor (CNI) should be personalized based on the balance between the risk of cancer progression and the risk of rejection (18). It is imperative to distinguish patients with heightened immunological risk, as they face a greater likelihood of graft loss stemming from rejection if CNI withdrawal occurs. Conversely, individuals exhibiting less aggressive immunological profiles and lower rejection risks may find potential benefits in CNI withdrawal or reduction, particularly when managing tumors with a high risk of progression. In certain cases, incorporating mTOR inhibitors into the immunosuppressive regimen appears promising for enhancing patient safety, graft survival, and oncological outcomes. In our study, the following modifications were made in the immunosuppressive therapy, namely, drugs were reduced, the dosage of calcineurin inhibitor and mycophenolate mofetil (MMF) were decreased, or MMF was discontinued. In nine patients, such modifications were made. In two patients, mTOR inhibitors were added to the previously prescribed drugs. In the other two patients, MMF was omitted. Cyclosporin A and Azathioprine were omitted in one patient each. A calcineurin inhibitor was substituted with an mTOR inhibitor in one patient. Tacrolimus and MMF were substituted with Azathioprine in one patient.

## Conclusions

Post-transplant RCC of native kidneys has a higher incidence. Vigilance and insight are necessary for practicing urologists to address this entity better. Meticulous follow-up is necessary for renal transplant patients and we recommend yearly screening ultrasound of native kidneys which would aid in the early detection of tumors and thereby their appropriate timely management. Early-stage RCC of the native kidney has comparable overall and disease-free survival rates as RCC in non-transplant patients. Judicious use of immunosuppressive agents and necessary post-nephrectomy modifications are suggested.
